# Axial Length Asymmetry in Patients with Unilateral Retinal Detachment

**DOI:** 10.3390/medicina62030514

**Published:** 2026-03-10

**Authors:** Hanru Wang, Jee Yao Loke, Weng Onn Chan, Stewart Lake

**Affiliations:** 1 Department of Ophthalmology, Flinders Medical Centre, Bedford Park, SA 5042, Australia; 2 Department of Ophthalmology, The Queen Elizabeth Hospital, Woodville South, SA 5011, Australia

**Keywords:** retinal detachment, retinal tear, axial length

## Abstract

*Background and Objectives:* Increased axial length is a major risk factor for retinal detachment. This study explored whether, in individuals presenting with unilateral disease, the longer eye was more likely to have rhegmatogenous disease. *Materials and Methods:* A consecutive cohort of 276 patients (100 female; mean age 62 ± 10 years), presenting with PVD-related retinal detachment or retinal tear between 2018 and 2024 in South Australia, had their axial length measured by partial coherence interferometry. Two-sample and paired *t*-tests were used to compare the affected and fellow eyes. *Results:* There was no significant difference in the axial length between the retinal detachment eyes and fellow eyes overall (n = 181; mean AL 24.93 vs. 24.82 mm; *p* = 0.41), or when considering a paired analysis of those with bilateral data (n = 139, 24.69 vs. 24.71 mm; *p* = 0.92), with the affected eye longer in 71/139 cases. Similar results were observed for retinal tear cases (n = 80; *p* = 0.20) and for the entire cohort with bilateral data (n = 219, *p* = 0.50). Sub-group analyses by axial length asymmetry (>0.1–1.0 mm, *p* = 0.63–0.97) and considering only larger eyes also found no significant difference (*p* = 0.38–0.98). *Conclusions:* Within individuals, the longer eye is not more likely to present with retinal detachment or retinal tear. This suggests that axial length is associated with, but not causative of, rhegmatogenous disease within individuals.

## 1. Introduction

Rhegmatogenous retinal detachment (RD) is a sight-threatening emergency, after which more than half are left with permanent vision impairment even with successful intervention. Risk factors associated with the development of RD include myopia, lattice degeneration, previous intraocular surgery, trauma, increased age, and a family history of RD [1,2]. Myopia and high myopia produce a 3-fold and 39-fold higher incidence of RD, compared to non-myopes [3]. Increased axial length (AL), the primary cause of myopia, is itself a strong risk factor linked to an increased incidence of RD, with RD eyes generally longer and in younger individuals than posterior vitreous detachment (PVD) eyes [1,2,4]. This study explores whether, in an individual, the AL has an impact on the eye presenting with rhegmatogenous disease after PVD. Understanding this relationship could aid in early detection and risk stratification, and potentially support the development of screening protocols.

## 2. Materials and Methods

The study was undertaken at retinal specialist services in Adelaide, South Australia. An observational consecutive cohort of patients was recruited, who presented with unilateral PVD-related retinal tear(s) or retinal detachment between 2018 and 2024 and consented to image capture and analysis for research. Excluded were those that presented with bilateral disease or with a history of rhegmatogenous disease in the fellow eye, and those with non-PVD-related retinal breaks. The study was approved by the Southern Adelaide Local Health Network (LNR 31.17) and Royal Australian and New Zealand College of Ophthalmologists (110.20) ethics committees. Informed consent was obtained from all patients prior to recruitment into the study. Subjects had their AL measured by partial coherence interferometry either before vitrectomy for retinal detachment, or after surgery if the macula was detached, no scleral buckle was performed, and pre-operative calculation was unreliable. Two-sample *t*-tests were used to compare AL between the affected and fellow eyes for the entire group, with paired *t*-tests performed comparing the affected to their fellow eyes in sub-groups with bilateral AL measurement.

## 3. Results

There were 276 patients (100 female) with a mean (standard deviation (SD)) age of 62 (10) years who had unilateral retinal detachment or retinal tears. There was no significant difference in the AL between the eye presenting with a retinal detachment and the fellow eye (FE) when considering all of the retinal detachment eyes (n = 181, mean (SD) AL 24.93 (1.32) mm (RD), 24.82 (1.24) mm (FE), *p* = 0.41, two-sample *t*-test), or considering only those eyes with bilateral axial length measurements (n = 139, RD eye AL = 24.69 (1.30) mm, FE AL = 24.71 (1.26) mm, 95% confidence intervals (CIs) −0.08, 0.09, paired *t*-test, *p* = 0.92), among whom in 71 of the 139 (51%), the eye with the RD was longer. There was no significant difference in AL between the retinal tear eyes and their contralateral eye (n = 80, RT AL = 24.59 mm (1.25) mm, FE AL = 24.53 (1.29) mm, paired *t*-test, *p* = 0.20) in whom 43 out of 80 (54%) had the retinal tear in the longer eye. Furthermore, there was no significant difference in axial length between the affected and fellow eyes combining all individuals with bilateral data and a retinal detachment or retinal tear (n = 219, paired *t*-test *p* = 0.50, 95% CI −0.05, 0.08), with the pathological eye longer in 114 of the 219 (52%) patients.

To assess whether axial length increased the risk of disease in patients with more asymmetric eyes, an analysis was performed considering only those eyes with AL asymmetry greater than 0.1 to 1.0 mm in 0.1 mm increments. Again, there were no significant differences in axial length between the retinal detachment and fellow eyes (*p* = 0.63–0.97; see Table 1).

Finally, there remained no significant difference in axial length between the affected eye and fellow eye when the analysis was confined to only those patients where the affected eye was longer than 23–26 mm (*p* = 0.38–0.98, Table 2).

## 4. Discussion

In this series of patients presenting with unilateral PVD-related rhegmatogenous disease, the affected eye was not longer than the fellow eye in paired, unpaired, whole, and sub-group analyses, and when considering escalating asymmetry or only longer eyes. This differs from the increased inter-individual risk seen when comparing myopes to non-myopes.

The relationship between RD and myopia is greater in the younger age group. The proportion of RD patients with high myopia (AL 26 ≥ mm) has been reported to be significantly higher in individuals younger than 50 years compared to those 50 and above (51.9% vs. 15.0%, *p* < 0.001), which is likely to be influenced by the younger group including those with non-PVD-related myopic retinal holes [5]. This supports the hypothesis that axial length may play a smaller role in RD development among the older age group with PVD-related disease.

## 5. Conclusions

If a greater axial length is a direct cause of retinal detachment, it would be expected to increase individual eye risk within individuals. The lack of any such sign in this series suggests that the relationship is an association, not a causative one, and that it is other features of myopia that lead to the increased risk. While this distinction may seem small, it will have importance in prediction and risk stratification models.

## Figures and Tables

**Table 1:** Is the retinal detachment eye the longer one? Patients stratified by magnitude of axial length asymmetry.

Minimum AL Asymmetry (mm)	Longer RD Eye, n (%)	Number of Patients	AL, RD vs. FE, Paired *t*-Test
0.0	71 (51)	139	0.92
0.1	46 (49)	93	0.97
0.2	35 (57)	61	0.75
0.3	28 (62)	45	0.70
0.4	17 (55)	31	0.94
0.5	11 (48)	23	0.70
0.6	10 (45)	22	0.63
0.7	9 (47)	19	0.72
0.8	8 (57)	14	0.95
0.9	7 (64)	11	0.80
1.0	2 (33)	6	0.73

AL = axial length, RD = retinal detachment, FE = fellow eye.

**Table 2:** Axial length difference between affected and fellow eye in larger eyes.

Affected Eye Minimum AL, (mm)	Longer Affected Eye, n (%)	Number of Patients	AL, Affected vs. FE, Paired *t*-Test
23.00	105 (52)	202	0.98
24.00	77 (52)	149	0.44
25.00	41 (54)	76	0.44
26.00	16 (52)	31	0.38

Affected eye = retinal detachment or retinal tear, AL = axial length, FE = fellow eye.

## Data Availability

The datasets used and/or analysed during the current study are available from the corresponding author on reasonable request.

## Abbreviations

The following abbreviations are used in this manuscript:

RD	Rhegmatogenous retinal detachment
RT	Retinal tear
AL	Axial length
PVD	Posterior vitreous detachment
SD	Standard deviation
FE	Fellow eye
